# Chronic IL-15 Stimulation and Impaired mTOR Signaling and Metabolism in Natural Killer Cells During Acute Myeloid Leukemia

**DOI:** 10.3389/fimmu.2021.730970

**Published:** 2021-12-17

**Authors:** Berna Bou-Tayeh, Vladimir Laletin, Nassim Salem, Sylvaine Just-Landi, Joanna Fares, Raphael Leblanc, Marielle Balzano, Yann M. Kerdiles, Ghislain Bidaut, Olivier Hérault, Daniel Olive, Michel Aurrand-Lions, Thierry Walzer, Jacques A. Nunès, Cyril Fauriat

**Affiliations:** ^1^ Aix-Marseille Université UM105, Centre National de la Recherche Scientifique (CNRS) UMR7258, Inserm UMR1068, Institut Paoli-Calmettes, Cancer Research Center of Marseille (CRCM), Marseille, France; ^2^ IBiSA Immunomonitoring Platform, Institut Paoli-Calmettes, Cancer Research Center of Marseille (CRCM), Marseille, France; ^3^ Aix-Marseille Université, Centre National de la Recherche Scientifique (CNRS), Institut National de la Santé et de la Recherche Médicale (INSERM), Centre d'Immunologie de Marseille-Luminy (CIML), Marseille, France; ^4^ Cibi Technological Platform, Cancer Research Center of Marseille (CRCM), Marseille, France; ^5^ Centre National de la Recherche Scientifique (CNRS) UMR 7292, LNOx Team, François Rabelais University, Tours, France; ^6^ Centre International de Recherche en Infectiologie (CIRI), Inserm U1111, Ecole Normale Supérieure de Lyon, Université Lyon 1, CNRS UMR5308, Lyon, France

**Keywords:** natural killer cells, acute myeloid leukemia, IL-15/mTOR signaling, metabolism, chronic stimulation, exhaustion

## Abstract

Natural Killer (NK) cells are potent anti-leukemic immune effectors. However, they display multiple defects in acute myeloid leukemia (AML) patients leading to reduced anti-tumor potential. Our limited understanding of the mechanisms underlying these defects hampers the development of strategies to restore NK cell potential. Here, we have used a mouse model of AML to gain insight into these mechanisms. We found that leukemia progression resulted in NK cell maturation defects and functional alterations. Next, we assessed NK cell cytokine signaling governing their behavior. We showed that NK cells from leukemic mice exhibit constitutive IL-15/mTOR signaling and type I IFN signaling. However, these cells failed to respond to IL-15 stimulation *in vitro* as illustrated by reduced activation of the mTOR pathway. Moreover, our data suggest that mTOR-mediated metabolic responses were reduced in NK cells from AML-bearing mice. Noteworthy, the reduction of mTOR-mediated activation of NK cells during AML development partially rescued NK cell metabolic and functional defects. Altogether, our data strongly suggest that NK cells from leukemic mice are metabolically and functionally exhausted as a result of a chronic cytokine activation, at least partially IL-15/mTOR signaling. NK cells from AML patients also displayed reduced IL-2/15Rβ expression and showed cues of reduced metabolic response to IL-15 stimulation *in vitro*, suggesting that a similar mechanism might occur in AML patients. Our study pinpoints the dysregulation of cytokine stimulation pathways as a new mechanism leading to NK cell defects in AML.

## Introduction

Acute myeloid leukemia (AML) is a group of hematological malignancies defined by the proliferation of abnormally differentiated myeloid blasts (1). Despite approval of several new drugs for AML treatment since 2017, the main treatment for AML patients remains chemotherapy (2, 3). Some patients can also benefit from treatment with hematopoietic stem cell transplantation (HSCT) which offers the best chance to cure AML (4). Success of AML treatment by HSCT indicates that this pathology is immunoresponsive. Particularly, AML cells are sensitive to the defense mediated by Natural Killer (NK) cells during haploidentical-HSCT (5).

NK cells are cytotoxic lymphocytes that can be activated in an antigen independent manner (6, 7). In both humans and mice, NK cells arise from a common innate lymphoid progenitor (CILP) and undergo several steps of differentiation, tightly regulated by a transcriptional program, and characterized by loss or acquisition of specific maturation markers (6, 8, 9). In mice, three main maturation subsets of NK cells can be defined based on the expression of CD27 and CD11b as follows: CD27^+^CD11b^—^, CD27^+^CD11b^+^ and CD27^—^CD11b^+^ from less to most mature (10). Although the development and maturation of NK cells start in the bone marrow (BM), NK cells circulate and populate a wide variety of tissues where they exert immune survey against cancer and virus-infected cells (6, 8, 11, 12). Besides their cytotoxic functions, NK cells can activate other actors of the innate and adaptive immunity by the secretion of cytokines such as IFN-γ (11, 13).

NK cell survival, maturation and activation rely on several cytokines, including interleukin (IL)-15 (14–16). IL-15 is an “IL-2-like” cytokine that binds to a trimeric receptor complex composed of the β chain of the IL-2/15R (CD122), the common gamma chain γc (CD132), and the α chain specific for IL-15 (CD215) (16). IL-15 in complex with IL-15Rα can be presented by hematopoietic or non-hematopoietic cells to neighboring NK cells expressing CD122 and CD132 (17–20). Upon binding to its receptor, IL-15 activates the same signaling pathway as IL-2, the JAK1-3/STAT5 signaling pathway. This leads to the expression of the STAT5 target genes and the maintenance of NK cell survival and proliferation (21, 22). IL-15 also induces the expression of proteins belonging to the family of suppressor of cytokine signaling (SOCS), such as CIS and SOCS1-3, which negatively regulate the activation of the JAK/STAT pathway (23, 24). Furthermore, it has been shown that high levels of IL-15 can stimulate NK cell metabolism by activating the mammalian target of rapamycin (mTOR) pathway (15, 25, 26). This metabolic response supports NK cell maturation and peripheral activation (15, 25–27). Hence, the perturbation of NK cell metabolism has been associated with altered NK cell functional responses in chronic diseases (28).

NK cells play a major role in the defense against AML cells. However, numerous studies, including ours, have reported defects in NK cells in AML patients which all severely impair the anti-tumor functions and affect the clinical outcomes (29–35). These defects include alteration of maturation, reduced cytotoxic functions, downregulation of triggering receptor expression or up-regulation of inhibitory/regulatory receptors, or down-regulation of transcription factors important for NK cell functions (29, 30, 36, 37). So far, the mechanisms underlying these alterations remain elusive, which hampers the development of efficient therapeutic strategies for the potentiation of NK cell functions in AML.

Leukemogenesis occurs in the BM, modifying the microenvironment to support the pathogenic process at the expense of normal hematopoiesis (38). AML development alters the cytokine expression profile in the BM (39, 40) and in the serum of patients (41). Although the impact of different cytokines on NK cell behavior is well known, how AML-induced modifications in these cytokines affect NK cell homeostasis and functions in AML has not been addressed. Here, we have used a mouse model of AML to investigate this point. In line with observations made in AML patients, we found that NK cell maturation and functions are altered in leukemic mice. We noted an active pro-inflammatory cytokine signaling in NK cells from leukemic mice. We suggest that chronic activating signaling, at least in part by IL-15, is a potential factor contributing to NK cell alterations in leukemic mice, by inducing defects in NK cell signaling and the exhaustion of NK cell functions and metabolism. Finally, we were able to show that NK cells of AML patients exhibit cues of metabolic defects, suggesting that similar mechanisms might occur in AML patients.

## Material and Methods

### Mice

Female CD45.2 C57BL/6J mice were purchased from Janvier labs (France). Male CD45.1 C57BL/6 mice were obtained from laboratory animal facility. *Ncr1* ^iCre^ mice were provided by Pr. E. Vivier (42) and were crossed with *TGFβRII* ^fl/fl^ mice provided by Dr. G. Guasch (43, 44) to produce *Ncr1* ^Cre/WT^ *TGFβRII* ^fl/fl^ mice. All mice were bred and maintained in the Cancer Research Center of Marseille animal facility under specific pathogen-free conditions. All mice were used between 6 and 12 weeks of age. In some experiments, mice were treated intraperitoneally with 100µg Poly(I:C) (*In vivo*gen) and sacrificed 16 hours later.

All animal protocols were performed in strict accordance with the European Directive 2010/63/EU on the use of animals for scientific purposes (agreement No. 2016041809182209) and were approved by an Institutional Animal Care and Use Committee.

### Leukemia Models

Two AML cell lines were used in our experiments, FLB1 cells provided by Prof. O. Hérault (45) and C1498 cells purchased from ATCC. Both leukemia cell lines are syngeneic to C57BL/6 mice. Freshly thawed 50 000 FLB1 cells (CD45.1) were resuspended in PBS and injected intravenously in congenic CD45.2 mice. For C1498, cells were expanded in DMEM medium supplemented with 10% Fetal Calf Serum (FCS) (Life Technologies) for one week. 25 000 C1498 cells (CD45.2) were then resuspended in PBS and injected intravenously in CD45.1 congenic mice. In all cases, AML development was monitored by flow cytometry analysis of the percentage of CD45.2 or CD45.1 positive cells in the blood. Control mice consisted of PBS injected mice.

In addition to transplantable models, inducible MLL-AF9 (iMLL-AF9) leukemic mice were kindly provided by Prof. J. Schwaller (Basel, Switzerland). Briefly the leukemia was induced by addition of 0.4mg/mL doxycycline in the drinking water until the appearance of leukemia as measured by white blood cell (WBC) count and a May-Grünwald-Giemsa staining (46). Mice were sacrificed when WBC count was four- to six-fold that of normal control mice. Control mice consisted of iMLL-AF9 transgenic mice not treated by doxycycline or mice having the doxycycline promoter but without the MLL-AF9 transgene (46).

### Treatment of Mice With Rapamycin

FLB1 cells were injected in mice and the percentage of leukemia cells in the blood was monitored by flow cytometry as previously described. Control mice were injected with PBS. Treatment of leukemic and control mice with rapamycin or with vehicle was started when the percentage of FLB1 cells in leukemic mice reached 0.5-2% of total CD45^+^ cells in the blood, which corresponds to 17 days post-injection. Rapamycin (R706203), dissolved in DMSO to a final concentration of 20 mg/mL and further diluted in 10% PEG-400/87,5% H2O, was daily injected intraperitoneally in mice to a final dosage of 3 mg/kg. Treatment of leukemic and control mice with rapamycin or with vehicle was stopped when the percentage of FLB1 cells in leukemic mice reached 10-20% of total CD45^+^ cells in the blood. Mice were then sacrificed immediately, or kept for 24h then treated intraperitoneally with 100µg Poly(I:C) (*In vivo*gen) and sacrificed 16 hours later (**Figure 6A**).

### Human Samples

All patient studies were carried out in accordance with the declaration of Helsinki with the agreement of the Institut Paoli-Calmettes institutional review board and all patients gave informed consent. All patients were diagnosed with AML and peripheral blood samples were taken and frozen by the institutional biobank (agreement N° LAM-NK2020-IPC 2020-019–20-001). Frozen samples were selected irrespective of AML subtypes. Healthy donor samples were provided by the local blood bank (Etablissement Français du sang) and frozen until use.

### Flow Cytometry

For murine cell phenotyping, Single-cell suspensions were prepared from BM, spleen and peripheral blood and were depleted of red blood cells using 1X RBC lysis buffer (eBioscience). For extracellular staining, cells were incubated with appropriate antibodies diluted in 2.4G2 supernatant, to block non-specific antibody binding, for 30 min at room temperature. Intracellular staining for IFN-γ was performed using BD Cytofix/Cytoperm™ Fixation/Permeabilization kit (BD Biosciences). Intracellular staining of Eomes, T-bet, Ki-67, Perforin, and Granzyme B was performed using FoxP3 Fixation/Permeabilization kit (eBioscience). For pSTAT5 and pS6 measurements, BM cells were prepared and cultured with murine IL-15 (mIL-15, eBioscience) at the indicated concentrations for one hour at 37°C. Intracellular staining of phosphorylated proteins was performed using the PerFix staining kit (Beckman Coulter). For absolute counting of cells, 10µL of CountBright absolute counting beads (ThermoFisher Scientific) were added to the stained cells just before acquisition on the flow cytometer.

For human NK cell phenotyping, frozen PBMCs were thawed and counted. Cells were then stained immediately or after 48h of culture at 37°C in RPMI medium (Gibco) supplemented with 10% FCS (Life Technologies), with or without 20ng/mL recombinant human IL-15 (Miltenyi Biotec). Extracellular staining was performed by incubating cells with antibodies for 30 min at RT. Intracellular staining of Eomes and T-bet was performed using FoxP3 Fixation/Permeabilization kit (eBioscience).

For human and murine NK cell phenotyping, acquisition was performed by a BD LSRII-SORP flow cytometer or a BD Fortessa flow cytometer (BD Biosciences). Data analysis was conducted with FlowJo software (BD). A list of all antibodies used in this study can be found in **Supplemental Table 1**.

### *In Vivo* Proliferation Assay

Control or leukemic mice were treated with two intraperitoneal injections of 100µg EdU (Invitrogen) 12 hours apart. Thirty-six hours after the second injection, mice were sacrificed and single cell suspensions were prepared from BM and labelled with antibodies against surface antigens. To measure EdU incorporation, cells were fixed and stained with Click-it Plus EdU flow cytometry kit (Invitrogen) according to manufacturer’s instructions.

### Apoptosis Detection by Annexin-V and 7-AAD Staining

Cells labelled with antibodies specific for surface proteins were incubated for 15 min at room temperature in the dark with Annexin-V (BioLegend) and 7-AAD (BD Biosciences) diluted in Annexin-V binding buffer (BioLegend). Cells were then immediately analyzed by flow cytometry. Apoptotic cells are defined as total Annexin-V positive cells.

### Killing Assay

NK cells were sorted from the spleen of leukemic or control mice using mouse NK cell isolation kit (StemCell, 19855) and FACS Aria II (BD Biosciences). Target YAC-1 cells or FLB1 cells were stained with 4µM of Cell Proliferation Dye eFluor™ 670 (Life Technologies) according to manufacturer’s instructions. Sorted NK cells were then incubated with target cells for four hours at 37°C at different effector to target (E:T) ratios. Target cell killing was measured using CellEvent™ Caspase-3/7 Green Detection Reagent (Life technologies) and analyzed by flow cytometry.

### Degranulation Assay

One million splenic cells of mice pre-stimulated or not with Poly(I:C) were cultured alone, or with 200 000 YAC-1 target cells, or FLB1 or C1498 leukemia cells, or stimulated with 200ng/mL PMA and 1µg/mL ionomycin (Sigma). After four hours of incubation at 37°C in the presence of anti-CD107a antibody, golgi stop and golgi plug (BD Biosciences), cells were stained and the percentages of NK cells positive for CD107a and IFN-γ were measured by flow cytometry. In some experiments, FLB1 leukemia cells were magnetically depleted from the spleen of leukemic mice by means of anti-CD45.1-biotin antibody (eBioscience) staining and EasySep™ Mouse Streptavidin RapidSpheres™ Isolation Kit (StemCell).

### Quantitative RT-PCR

RNA was isolated from total BM cells of leukemic or control mice using the RNeasy mini kit (Qiagen). cDNA was then obtained using the SuperScript II Reverse Transcriptase (Invitrogen). Quantitative PCR was performed using *TGF-β1* and *Gapdh* (internal control) TaqMan assays (Applied Biosystems) and a CFX96 real-time PCR detection system (Bio-Rad). Gene expression was normalized to internal control (ΔCt = Ct gene of interest–Ct internal control) and relative mRNA expression to GAPDH was calculated as 2^–ΔCt^.

### RNA Sequencing

BM cells from five mice per group (control or FLB1 injected) were pooled per experiment prior to NK cell sorting, and three independent experiments were performed. NK cells were first sorted using EasySep™ Mouse NK Cell Isolation Kit (STEMCELL Technologies), then with Aria II on the basis of live/CD3^-^CD19^-^NK1.1^+^CD27^+^CD11b^—^ gating. RNA was isolated from sorted cells using the RNeasy micro plus kit (Qiagen) and mRNA quality was evaluated using an Agilent 2100 (Pico Chip). RNA Sequencing was performed by the GenomEast platform, a member of the ‘France Génomique’ consortium (ANR-10-INBS-0009). RNA-Seq analysis was done with a production pipeline, as follows. Quality control was done with FastQC and sequence alignment against mm10 genome with Subread 1.6. Quality control revealed an optimal quality level (Phix) and a high number of aligned reads. Alignment was done in pair-read mode with removal of multiple-aligned reads. Gene count was performed with featureCount (SubRead package). Further analysis of gene counts (Differential analysis of gene expression) was done using R with EdgeR package. Count normalization was done with TMM method (EdgeR). Analysis of differential expression was performed with Limma on the web-based application Phantasus (https://bioconductor.org/packages/release/bioc/html/phantasus.html or https://artyomovlab.wustl.edu/phantasus/). Analysis of enriched pathways was first performed using EnrichR (https://amp.pharm.mssm.edu/Enrichr/) (47). Finally, Gene set enrichment analysis (GSEA) was also performed, with the GSEA software from the Broad Institute, using the Hallmark Gene Sets from the molecular signature database v7.1 (https://www.gsea-msigdb.org/gsea/msigdb/index.jsp). The datasets presented in this study can be found in online repositories. The names of the repository/repositories and accession number(s) can be found below: GEO, GSE180409.

### Glucose Uptake Measurement

Freshly isolated BM or splenic cells were resuspended in glucose free RPMI (Gibco) supplemented with 100µM 2-NBDG (2-(N-(7-Nitrobenz-2-oxa-1,3-diazol-4-yl)Amino)-2-Deoxyglucose, Invitrogen) for 10 min et 37°C. Cells were then washed with PBS, stained with antibodies and analyzed by flow cytometry.

### Mitochondrial Mass and Mitochondrial Superoxide Measurements

Total mitochondrial mass or mitochondrial superoxide were measured by incubating cells for 10 min at 37°C with 500nM MitoTracker™ Deep Red (Life Technologies) or 5µM MitoSOX™ Red (Life Technologies) respectively. Cells were washed twice with PBS, stained with antibodies and analyzed by flow cytometry.

### Cytokine Measurements in BM Supernatants

Femurs and tibias, isolated from leukemic or control mice, were dissociated from adjacent muscles, cut in two, and centrifuged in RPMI medium. BM supernatants of 5 different leukemic or control mice were pooled and analyzed for 97 different mouse cytokines using mouse cytokine array C6 according to manufacturer’s instructions (Antibodies Online). The cytokine membranes were quantified by densitometric analysis using ImageJ.

For IL-15 measurement, BM supernatants of leukemic or control mice were analyzed using mouse IL-15 DuoSet ELISA (R&D systems) according to manufacturer’s recommendations.

### Statistical Analyses

Statistical analyses were performed using Prism software (GraphPad). For two data sets comparison, unpaired nonparametric Mann-Whitney *t*-test was used. For comparison of more than two data sets, unpaired nonparametric Kruskal-Wallis *t*-test was used. Levels of statistical significance are expressed as *P* values: *P< 0.05, ** *P <*0.01, *** *P <*0.001, **** *P <*0.0001. Error bars represent SEM.

## Results

### NK Cell Maturation and Homeostasis Are Altered in Mice Bearing Acute Myeloid Leukemia

To gain insight into the mechanisms underlying NK cell defects observed in AML patients, we used FLB1 cells as a syngeneic mouse model of AML. This model is based on the overexpression in hematopoietic stem cells of *hoxa9* and *meis1* genes that are commonly overexpressed in AML (48, 49). FLB1 cells are of myeloid origin and phenotype (data not shown) and almost recapitulate human AML (45). They are recognized and killed by syngeneic NK cells *in vitro*, although to a lesser extent than the classical murine NK target cell YAC-1 (**Supplemental Figure 1A**). Intravenous injection of FLB1 cells in syngeneic mice resulted in a progressive bone marrow (BM), blood and spleen invasion (**Supplemental Figure 1B**). Following AML development, NK cell frequency was increased in leukemic mice BM (**Supplemental Figure 1C**), albeit a significant decrease was observed in non-leukemic and NK cell numbers (**Supplemental Figures 1D, E**). There was no significant change in the percentage nor in the number of NK cells in the spleen of leukemic mice compared to control mice (**Supplemental Figures 1C–E**).

**Figure 1:**
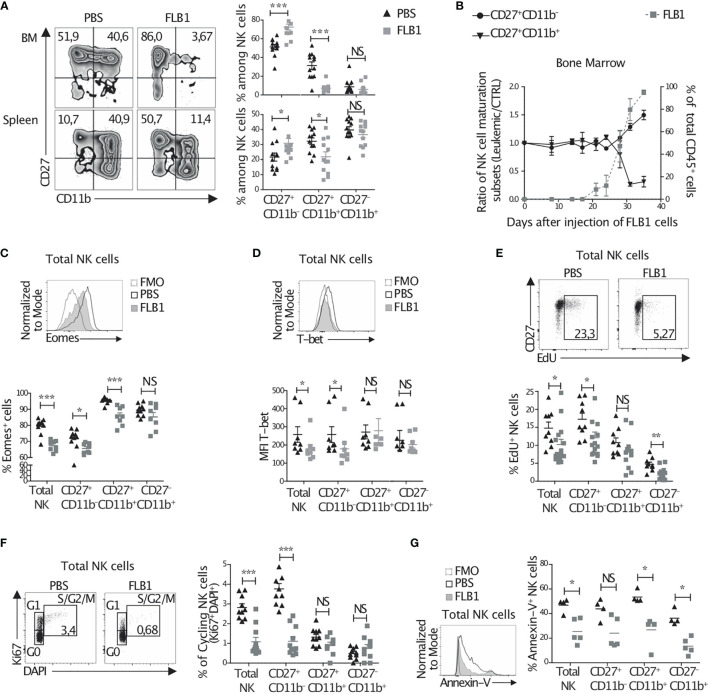
NK cell maturation and homeostasis are altered in leukemic mice. **(A–D)** Freshly isolated cells from the bone marrow (BM) or the spleen of leukemic (FLB1 injected) or control (PBS injected) mice were stained and analyzed by flow cytometry. **(A)** Left: the percentages on zebra plots indicate the CD27^+^CD11b^—^ and CD27^+^CD11b^+^ NK cell populations in relation to total NK1.1^+^CD3^-^CD19^-^ cells. Right: the percentages of all three NK cell maturation subsets, as determined by CD27 and CD11b expression, in the BM (upper graph) and spleen (bottom graph) (n=12 mice/group in four independent experiments). **(B)** The percentages of CD27^+^CD11b^—^ and CD27^+^CD11b^+^ NK cell maturation subsets (left Y axis) and of CD45.1^+^ FLB1 cells (right Y axis) evaluated by flow cytometry in the BM at the indicated days after FLB1 injection (n=2-6 mice/group in two independent experiments). **(C)** The percentage of Eomes positive cells in relation to total NK cells and NK cell maturation subsets in the BM of leukemic or control mice (n=8-10 mice/group in two independent experiments). **(D)** The level of expression of T-bet in total NK cells and NK cell maturation subsets in the BM of leukemic or control mice (n= 8 mice/group in three independent experiments). **(E, F)** Leukemic or control mice were injected twice with 100μg of EdU 12h apart. 36h after the second injection of EdU, mice were sacrificed and BM cells were extracted and stained to measure EdU incorporation. Cells were also stained with DAPI and anti-NK1.1, -CD3, -CD19, -CD27, -CD11b and -Ki67 antibodies and were analyzed by flow cytometry. **(E)** The percentage on dot plots indicate the percentage of EdU incorporation in total NK cells of leukemic and control mice. The graph represents the percentage of EdU positive NK cells and NK cell maturation subsets. **(F)** The percentage on dot plots indicate the percentage of NK cells Ki67^+^DAPI^+^ in S/G2/M phase or cycling NK cells. The graph represents the percentage of cycling cells of total NK cells and of the three NK cell maturation subsets (n=10-11 mice/group in three independent experiments). **(G)** BM cells freshly isolated from control and leukemic mice were stained and analyzed by flow cytometry to determine the percentage of apoptotic cells defined as total Annexin-V(AnnV)^+^ cells (n=4-5 mice/group in three independent experiments). The values are presented as the mean +/- SEM. *p < 0.05, **p < 0.01, ***p < 0.001, NS, Non Significant, as determined by Mann-Whitney test.

We next analyzed NK cell maturation by means of CD27 and CD11b expression. We observed an increase in the percentage of the immature CD27^+^CD11b^—^ NK cell subset and a sharp decrease in the percentage of mature CD27^+^CD11b^+^ NK cells in the BM and spleen of leukemic mice (**Figure 1A**), while no significant change could be observed for CD27^—^CD11b^+^ NK cell frequency (**Figure 1A**). With respect to numbers, all subsets were reduced in leukemic mice BM and not changed in the spleen, except CD27^+^CD11b^—^ immature NK cells which were increased in the spleen of leukemic mice (**Supplemental Figure 1F**). Moreover, we noted that NK cell maturation imbalance in the BM paralleled leukemia progression in this organ (**Figure 1B**). Finally, we evaluated NK cell maturation in other mouse models of AML: the murine C1498 transplantable model and the genetically induced model of *MLL-AF9* transgenic mice. Both tumors invade progressively the BM and induce AML-like diseases (46, 50). Similarly to the FLB1 model, we found a significant increase in the percentage of the immature CD27^+^CD11b^—^ NK cells in both leukemia models (**Supplemental Figure 1G**). These data suggest that the hypomaturation of NK cells is common in leukemic mice, and validate the use of our FLB1 injected mice as a surrogate model for the study of NK cell alterations in AML.

NK cell maturation is regulated by transcription factors, including T-bet and Eomesodermin (Eomes) (51). In particular, it has been shown that Eomes regulates NK cell maturation from CD27^+^CD11b^—^ stage to CD27^+^CD11b^+^ stage (52, 53). To assess whether Eomes and T-bet are dysregulated in NK cells from leukemic mice, we evaluated their expression in BM and spleen NK cells by flow cytometry. We observed a decrease in the percentage of Eomes-expressing NK cells in leukemic mice (**Figure 1C** and **Supplemental Figure 1I**). We also noted a decrease in the level of Eomes expression in Eomes^+^ NK cells, although this decrease is not statistically significant in the BM (**Supplemental Figure 1H, J**). Moreover, reduced expression of T-bet in NK cells from leukemic mice was found (**Figure 1D** and **Supplemental Figure 1K**), suggesting an altered expression of the transcription factors regulating NK cell maturation after leukemia progression in mice.

The hypomaturation of NK cells in leukemic mice could also originate from an increase in the proliferation rate of immature subsets and/or in the apoptosis of mature subsets. To address this, we first assessed *in vivo* homeostatic proliferation of NK cell subsets by means of EdU incorporation. We observed a decrease in the percentage of proliferating NK cells in the BM of leukemic mice compared to control mice (**Figure 1E**). This decrease was observed for all subsets, especially for CD27^+^CD11b^—^ NK cells (**Figure 1E**) and was confirmed by a measurement of Ki67 expression (**Figure 1F**). Moreover, *ex vivo* apoptosis analysis by measuring Annexin-V expression revealed a decreased apoptosis especially in mature CD11b^+^ subsets (**Figure 1G**). Altogether, these data suggest an altered homeostatic proliferation, apoptotic rate, and maturation in NK cells from leukemic mice.

### The Functions of NK Cells Are Altered in Leukemic Mice

**Figure 2:**
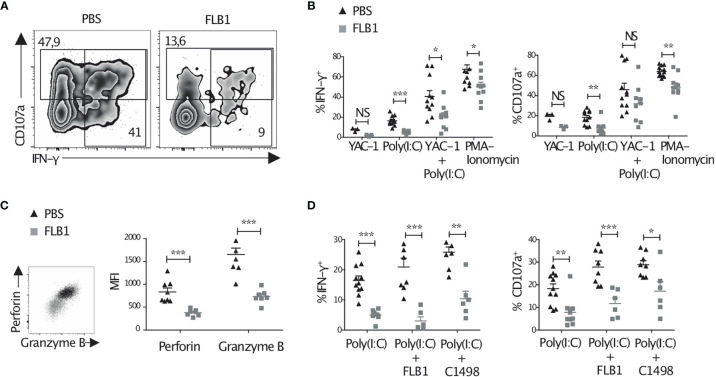
NK cell functions are altered in leukemic mice. Cells were freshly extracted from the spleen of leukemic or control mice pre-injected or not with 100μg Poly(I:C) 16h prior to sacrifice. Cells were then stained immediately **(C)**, or after four hours of culture alone, or with YAC-1/FLB1/C1498 cells, or with PMA and ionomycin, in the presence of Golgi-stop, Golgi-plug and anti-CD107a antibody **(A, B, D)**. Cells were then analyzed by flow cytometry. **(A)** Zebra plots show the level of CD107a exposure and IFN-γ production by total NK cells primed with Poly(I:C) and cultured with YAC-1 cells. **(B)** The graphs show the percentages of NK cell IFN-γ production and degranulation (CD107a exposure) in result to all stimulations (n=9-12 mice/group in at least three independent experiments, except for YAC-1: n=3 mice/group in one experiment). **(C)** The level of perforin and granzyme B production, as measured by MFI, by total NK cells after priming with Poly(I:C) (n=7-8 mice/group in two independent experiments). **(D)** The percentages of IFN-γ production and degranulation by NK cells primed with Poly(I:C) and cultured with FLB1 or C1498 AML cell lines (n=6-11 mice/group in at least two independent experiments). The values are presented as the mean +/- SEM. *p < 0.05, **p < 0.01, ***p <0 .001, NS, Non Significant, as determined by Mann-Whitney test.

Next, we compared the functions of splenic NK cells from leukemic and control mice. NK cells from leukemic mice tend to produce less IFN-γ and degranulate less than control NK cells in response to YAC-1 target cells (**Figure 2B**). Priming of NK cells *in vivo* with the TLR3 ligand poly(I:C), injected 16h prior to the functional analysis, improved their degranulation and cytokine production but these functions remained lower in NK cells from leukemic mice compared to controls (**Figures 2A, B**). *Ex vivo* stimulation with PMA and ionomycin, performed in order to measure the maximal functional potential of NK cells, also resulted in decreased degranulation and IFN-γ production in NK cells from leukemic mice (**Figure 2B**). A similar reduction of NK cell functions was observed when FLB1 cells were depleted from the spleen of leukemic mice before *ex vivo* stimulation, ruling out that the presence of leukemic cells during functional assays may be responsible for the observed effects (**Supplemental Figure 2A**). In line with the reduction of NK cell functions, NK cells from leukemic mice expressed lower levels of perforin and granzyme B (**Figure 2C**). Finally, we assessed the functions of NK cells from leukemic mice, primed with poly(I:C), in response to AML cell lines, FLB1 and C1498. Decreased degranulation and IFN-γ production by NK cells were observed as compared to controls (**Figure 2D**). Noteworthy, defective functions of NK cells in response to *in vivo/ex vivo* stimulation were independent of their maturation status (**Supplemental Figures 2B, C**). Altogether, these results show that NK cells in leukemic mice display a reduction of their effector functions.

### Activation of Cytokine Signaling and of the IL-15 Pathway in NK Cells From Leukemic Mice

**Figure 3:**
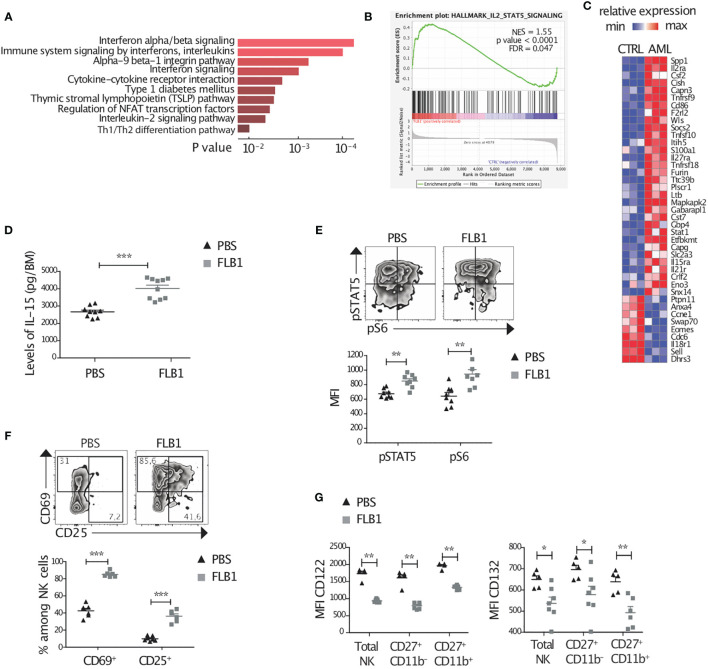
Active NK cell cytokine signaling and elevated levels of IL-15 in the bone marrow of leukemic mice. **(A–C)** CD27^+^CD11b^—^ NK cells were sorted from leukemic (FLB1 injected) or control (PBS injected) bone marrows (BMs) and RNA sequencing was performed. Data are representative of three independent experiments (five mice/group/experiment). **(A)** Enrichment pathway analysis showing up-regulated pathways in NK cells from leukemic mice compared to controls. **(B)** GSEA enrichment plot for the IL-2-STAT5 signaling pathway. **(C)** Heatmap representing expression of selected genes (IL-2/STAT5 signaling pathway (Reactome), for all genes: p<0.05, Log Expression>4). Color code corresponds to normalized (by row) minimum and maximum gene expression. **(D)** The levels of IL-15 in the BM supernatants of leukemic or control mice were measured by ELISA (n=9-10 mice/group). **(E–G)** Freshly isolated BM cells of control and leukemic mice were stained and analyzed by flow cytometry. **(E)** Zebra plots show the level of phosphorylation of STAT5 and S6 in representative leukemic or control BM-NK cells. The graphs show the MFI of intracellular pSTAT5 and pS6 by total NK cells (n=8 mice/group in two independent experiments). **(F)** Zebra plots show the expression of CD69 and CD25 in BM-NK cells from leukemic or control mice. The mean percentages of CD69 expressing or CD25 expressing NK Cells in the BM of leukemic and control mice are represented (n=6-7 mice/group in two independent experiments). **(G)** MFI of the surface expression of IL-15 receptors by total NK cells and by CD27^+^CD11b^—^ and CD27^+^CD11b^+^ NK cell maturation subsets (n=5-7 mice/group in two independent experiments). The values are presented as the mean +/- SEM. *p < 0.05, **p < 0.01, ***p < 0.001, as determined by Mann-Whitney test.

NK cell maturation, homeostasis, as well as activation, are critically dependent on cytokine priming. We hypothesized that NK cell defects in leukemic mice might stem from changes in cytokine signaling. To address this point, we first measured the modifications in the cytokine profile of the BM supernatants of leukemic and control mice using a mouse cytokine array for the quantification of 97 different cytokines. Several cytokines and soluble factors were differentially expressed (**Supplemental Figure 3A**). We noted an increase in the levels of some pro-inflammatory cytokines, such as Leptin, IFN-γ, CD27, CD30, hepatocyte growth factor (HGF), but also of some anti-inflammatory cytokines such as IL-10 and CTLA-4 or some chemokines such as CXCL1 and CCL21 (**Supplemental Figure 3A**). Next, we performed RNA sequencing analysis on the immature CD27^+^CD11b^—^ NK cells from the BM of leukemic and control mice. We used a web-based software (EnrichR) which provides the 10 most significantly enriched pathways (**Figure 3A**). Enrichment analysis revealed a strong response to different proinflammatory cytokines including type-I IFNs and Interleukin/IL-2 signaling in NK cells from leukemic mice. A further analysis of the RNA sequencing data using GSEA confirmed that the IL-2/IL-15 pathway was engaged in NK cells isolated from leukemic mice (**Figures 3B, C**). Amongst the genes from this pathway, *Cish*, known to be induced by IL-2 and IL-15, was significantly up-regulated (FC=1.9, adjusted p value = 0.02) (**Figure 3C**) (24).

IL-15 is one of the most important cytokines for NK cells. In particular, IL-15/mTOR pathway regulates NK cell maturation from CD11b^—^ to CD11b^+^ stage and facilitates NK cell activation in periphery (15, 54), two checkpoints that are altered in our leukemic mice. We first measured by ELISA the levels of IL-15 in the BM supernatants. Interestingly, concentration of IL-15, measured by ELISA, in the BM of FLB1-injected mice was higher compared to controls an increase in the levels of IL-15 in the BM of FLB1-injected mice compared to controls (**Figure 3D**). We also noted a tendency towards an increase in the levels of IL-15 in the BM of MLL-AF9 leukemic mice (**Supplemental Figure 3B**). Next, we measured *ex vivo* levels of phosphorylation of STAT5 (pSTAT5) and of the mTOR associated ribosomal protein S6 (pS6) in NK cells. We noted an increase in the amount of phosphorylated STAT5 and S6 in NK cells from leukemic mice compared to controls (**Figure 3E**), suggesting an activation of the signaling pathways downstream of IL-15 receptor. In line with an activated profile, flow cytometry analysis showed an increase in the percentage of NK cells expressing CD69 and CD25, two markers that can be induced by IL-15 (25, 55) (**Figure 3F**). Finally, we measured the expression of IL-15 receptor subunits on BM-NK cells. The β chain of the IL-2/IL-15 receptor (CD122) was downregulated in FLB1-injected and MLL-AF9 leukemic mice (**Figure 3G** and **Supplemental Figure 3C**). We also noted a decrease in the surface expression of the common γ chain (CD132) in NK cells from FLB1-injected mice, independently of the stage of NK cell maturation (**Figure 3G**). A total (intracellular and extracellular) *versus* extracellular staining of CD122 in NK cells showed that the whole surface and intracellular levels for this receptor were decreased in leukemic mice NK cells (**Supplemental Figure 3D**). The increase in the levels of IL-15 in the BM and the activation of the IL-15 signaling pathway in NK cells from leukemic mice strongly suggest that the downregulation of IL-15 receptor subunits is due to ligation to IL-15 and increase in its degradation (24, 56), in line with *cish* mRNA upregulation. Altogether, these data strongly suggest that NK cells are chronically stimulated by cytokines in the leukemic microenvironment and in particular by the pro-inflammatory cytokine IL-15.

### NK Cells of Leukemic Mice Are Hyporesponsive to IL-15 Stimulation *In Vitro*

**Figure 4:**
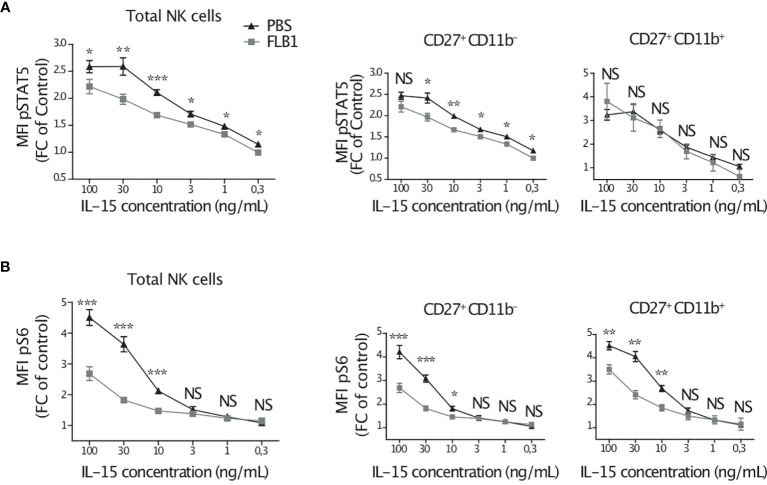
NK cells of leukemic mice are hyporesponsive to IL-15 stimulation *in vitro*. Bone marrow cells of leukemic (FLB1 injected) or control (PBS injected) mice were cultured with the indicated concentrations of IL-15 for one hour. Cells were then stained and analyzed by flow cytometry. MFI of intracellular pSTAT5 **(A)** and pS6 **(B)** for total NK cells and for CD27^+^CD11b^—^ and CD27^+^CD11b^+^ NK cell maturation subsets are given as a ratio of MFI at the indicated concentration of IL-15 normalized to the MFI in the absence of IL-15 (n=5-8 mice/group in two independent experiments). The values are presented as the mean +/- SEM. *p < 0.05, **p < 0.01, ***p < 0.001, NS, Non Significant, as determined by Mann-Whitney test.

Our data indicate that NK cells from leukemic mice are exposed to IL-15 *in vivo*. We wanted to assess the consequences of this exposure to their responsiveness to this cytokine *ex vivo*. For this, we cultured BM cells, from leukemic or control mice, with increasing concentrations of IL-15 for 1 hour, and we measured the expression of phosphorylated STAT5 and S6 in NK cells by flow cytometry. IL-15-induced phosphorylation of STAT5 was reduced in total and CD27^+^CD11b^—^ NK cells from leukemic mice compared to control mice, at all IL-15 concentrations tested (**Figure 4A**). The S6 phosphorylation was induced in NK cells only at high concentrations of IL-15 known to activate the mTOR pathway (15). Nonetheless, S6 phosphorylation was lower in NK cells from leukemic mice compared to control mice, and this reduction was independent of the maturation stage of NK cells (**Figure 4B**). These data indicate that NK cells from leukemic mice are hyporesponsive to additional short stimulation with IL-15 *in vitro*. Of note, culturing NK cells for a longer period of time (24h) with IL-15 increased their responsiveness to target cells but NK cells from leukemic mice were still poor responders compared to controls (data not shown), thus confirming that the responsiveness of NK cells to IL-15 stimulation *in vitro* was reduced in leukemic mice.

### NK Cells of Leukemic Mice Have Reduced Expression of Metabolic Markers

Chronic stimulation of NK cells with IL-15 is known to generate deleterious effects, assimilated to cell exhaustion, on NK cell functions and metabolism (55, 57–59). So far, our results suggested that BM-NK cells in leukemic mice bath in a proinflammatory milieu and in particular they may be chronically stimulated by IL-15 which leads to deregulation in the mTOR pathway. Therefore, we next wondered what would be the consequences of this exposure on NK cell metabolism. First, we measured by flow cytometry the size and granularity of NK cells *ex vivo* as simple surrogate markers of NK cell activation and metabolic response (15, 60). In contrast to the activation status, the size and granularity of BM-NK cells, but not that of spleen NK cells, tended to be lower in leukemic mice compared to control mice (**Supplemental Figure 4A**). We next measured the expression of the heavy chain of L-amino acid transporter SLC3A2 (CD98) and the transferrin receptor (CD71) *ex vivo*. Indeed, these markers are known to be upregulated in metabolically active NK cells (15, 60). Interestingly, the expression of CD98 was reduced, irrespective of maturation stages, in leukemic mice BM-NK cells compared to control mice (**Supplemental Figure 4A**). In addition, we observed a tendency towards a decrease in the expression of CD71 (**Supplemental Figure 4A**). These data indicate that despite their activation, NK cells of leukemic mice seem to be less metabolically active.

**Figure 5:**
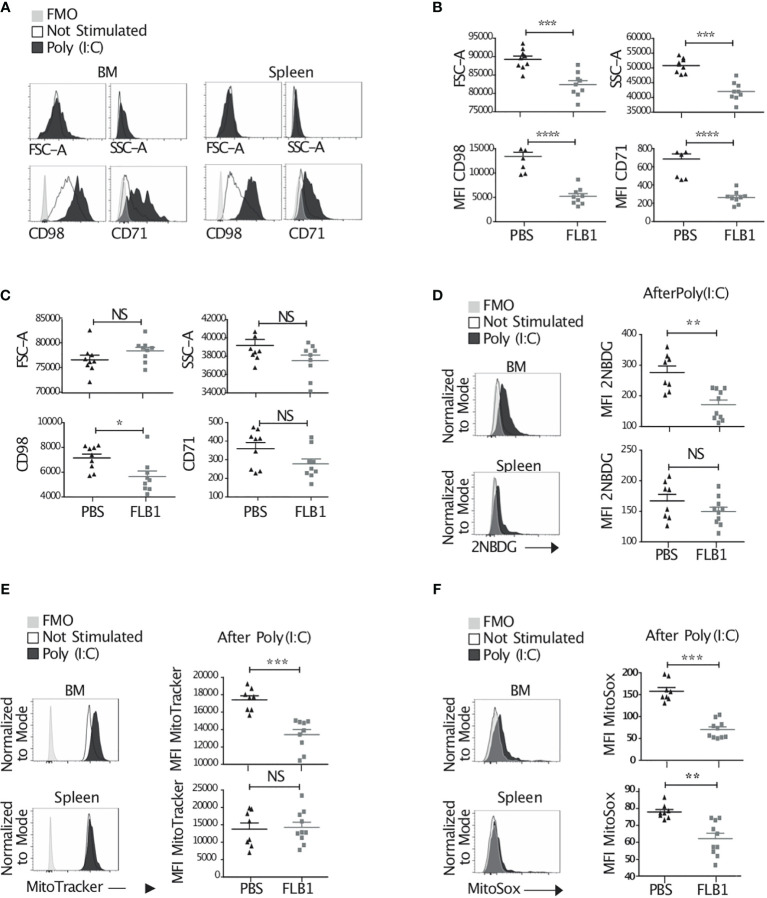
Reduced metabolic response and exhausted features by NK cells from leukemic mice. **(A–F)** Mice were injected or not with 100μg Poly(I:C) and sacrificed 16h later. Bone marrow (BM) and splenic cells were extracted, stained and analyzed by flow cytometry. **(A)** Histograms represent the size, granularity, and the expression of nutrient transporters by NK cells. **(B, C)** MFI of FSC-A, SSC-A, CD98 and CD71 by total BM **(B)** or splenic **(C)** NK cells of control (PBS injected) and leukemic (FLB1 injected) mice after Poly(I:C) injection (n=9 mice/group in two independent experiments). **(D–F)** Histograms represent the glucose uptake **(D)**, the mitochondrial mass **(E)** and the mitochondrial reactive oxygen species **(F)** by total BM and splenic NK cells of control mice not stimulated or stimulated with Poly(I:C) (Left panel). The graphs show MFI for 2NBDG **(D)**, MitoTracker **(E)** and MitoSox **(F)** expression by NK cells of control and leukemic mice after Poly(I:C) injection (right panel) (n=8-10 mice/group in two independent experiments). **(A, D, E, F)** Representative data of NK cells from control mice not stimulated (empty lines), stimulated with Poly(I:C) (black filled lines) and negative controls (FMO, grey shade). The values are presented as the mean +/- SEM. *p < 0.05, **p < 0.01, ***p < 0.001, ****p < 0.0001, NS, Non Significant, as determined by Mann-Whitney test.

*In vivo* injection of poly(I:C) is known to strongly enhance NK cell metabolism *via* IL-15 and the mTOR pathway (15, 60). Therefore, we next compared the metabolic responses of NK cells in leukemic and control mice after poly(I:C) injection 16h prior to sacrifice (**Figure 5A**). We observed a reduced NK cell size, granularity, and expression of nutrient transporters in BM-NK cells from leukemic mice (**Figure 5B** and **supplemental Figure 4B**). We also observed a tendency towards a decrease of these markers in spleen-NK cells from leukemic mice compared to controls (**Figure 5C** and **supplemental Figure 4B**). Moreover, we assessed the uptake of glucose by *ex vivo* NK cells primed with Poly(I:C) by measuring 2NBDG staining. We showed a reduced glucose uptake in BM-NK cells from leukemic mice compared to controls (**Figure 5D** and **Supplemental Figure 4C**). Finally, mitochondrial activity in NK cells, including mitochondrial mass and mitochondrial reactive oxygen species (ROS), measured by MitoTracker and MitoSOX staining, respectively, were also reduced in leukemic mice (**Figures 5E, F** and **Supplemental Figure 4D**). Altogether, these data suggest that NK cells from leukemic mice exhibit reduced metabolic response after activation of the mTOR pathway.

**Figure 6:**
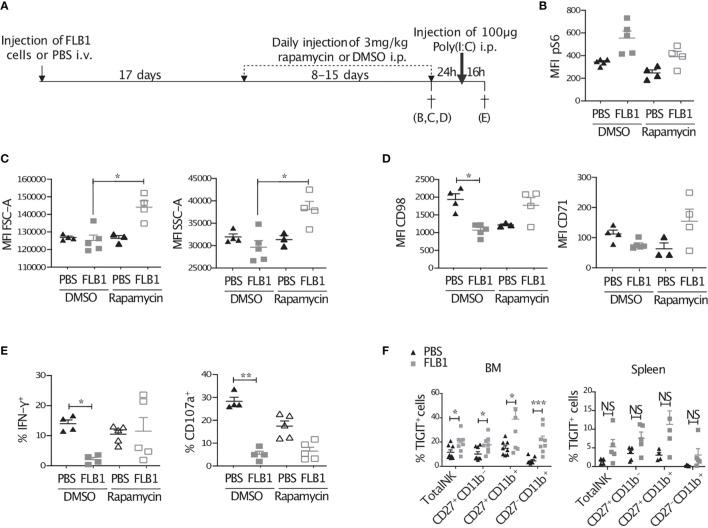
Treatment of mice with rapamycin improves the metabolic fitness and IFN-γ production of NK cells from leukemic mice. **(A)** Schematic representation of the protocol. Control (PBS injected) or leukemic (FLB1 injected) mice were treated with daily injection of rapamycin or DMSO for 8 to 15 days. The duration of the treatment was dependent of the leukemic progression in mice and was stopped when the percentage of FLB1 cells reaches 10-20% of total white blood cells in peripheral blood. Mice were then sacrificed **(B–D)**, or kept for 24h without treatment and then injected with 100 µg Poly(I:C) and sacrificed 16h later **(E)** (n=3-5 mice/group). **(B)** The graph represents the level of phosphorylation of S6, expressed as MFI, in bone marrow (BM) NK cells. **(C)** The graphs represent the size (FSC), granularity (SSC) of BM-NK cells. **(D)** The graphs represent the level of expression of nutrient transporters (CD98 and CD71) in BM-NK cells. **(E)** Freshly extracted splenocytes were stained and analyzed by flow cytometry. The graphs represent the percentage of NK cells expressing IFN-γ and the marker of degranulation CD107a. **(F)** Freshly isolated BM and splenic cells of leukemic or control mice were stained and analyzed by flow cytometry. The graphs show the percentage of NK cells and NK cell maturation subsets expressing TIGIT (BM: n=8 mice/group in three independent experiments; Spleen: n=4-5 mice/group). The values are presented as the mean +/- SEM. *p < 0.05, **p < 0.01, ***p < 0.001, NS, Non Significant as determined by Kruskal-Wallis test **(B–E)** or Mann-Whitney test **(F)**.

Our data suggest that IL-15/mTOR pathway is chronically stimulated in NK cells from leukemic mice which is associated with metabolic and functional defects. We hypothesized that the reduction of the activation of the mTOR pathway during AML progression might reduce these defects. Therefore, we treated mice with daily injections of rapamycin, an inhibitor of the mTOR complex 1 (mTORC1), during the exponential phase of the engraftment of AML cells in the organs (**Figure 6A** and **Supplemental Figure 1B**). First, we measured the levels of S6 phosphorylation in NK cells *ex vivo* and observed a decrease in leukemic mice treated with rapamycin compared to those treated with vehicle (**Figure 6B**). This increased S6 phosphorylation remained higher in NK cells of leukemic mice compared to control mice even in rapamycin treated group, indicating that the regimen of rapamycin treatment used in our experiments reduced, but did not completely abrogate, the activation of the mTOR pathway (**Figure 6B**). Next, we assessed NK cell metabolic markers such as the size, granularity and expression of nutrient transporters CD98 and CD71. We showed an increase in these markers in leukemic mice treated with rapamycin compared to mice treated with vehicle (**Figures 6C, D**). Finally, NK cell secretion of IFN-γ, but not their degranulation, tended to be higher in leukemic mice treated with rapamycin and activated with Poly(I:C) injection 16h prior to sacrifice compared to mice treated with vehicle (**Figure 6E**). Altogether, these data indicate that the reduction of the activation of mTOR during AML development partially rescued NK cell metabolic and functional defects.

### The Depletion of TGF-β Signaling Does Not Restore NK Cell Metabolic and Functional Defects in Leukemic Mice

TGF-β is a prominent immunosuppressive cytokine that can inhibit the metabolism and the functions of NK cells, in particular by repressing the mTOR pathway (61–63). TGF-β can also decrease the expression of the β subunit of IL-15 receptor leading to reduced IL-15 signaling (64). In our model we noted an increase in phosphorylated SMAD2/3 (pSMAD2/3) proteins (**Supplemental Figure 5A**) hinting at activation of TGF-β signaling as evidenced by an increase in phosphorylated SMAD2/3 (pSMAD2/3) proteins (**Supplemental Figure 5A**). Not surprisingly TGF-β mRNA levels also had a tendency to increase in BM cells of leukemic mice compared to control mice (**Supplemental Figure 5B**). Therefore, we wondered whether TGF-β could be involved in the alteration of NK cell IL-15 signaling, metabolism, and functions. To address this question, we used mice in which the TGF-βRII receptor was depleted in NK cells specifically (NK-TGFβRII-/- mice). When NK-TGFβRII^-/-^ mice were injected with FLB1 cells, we did not observe any restoration of NK cells defects, although phosphorylation of SMAD2/3 was completely abrogated (**Supplemental Figure 5C–G**). Hence, CD122 expression, STAT5 and S6 phosphorylation, degranulation, IFN-γ production, and expression of nutrient transporters remained reduced in NK cells from leukemic mice compared to controls (**Supplemental Figure 5D–G**), ruling out the implication of TGF-β in the alterations of NK cells in leukemic mice.

### NK Cells of Leukemic Mice Display Exhaustion Features

Thus far as our data suggest that NK cells in leukemic mice are chronically stimulated by IL-15 and exhibit defective homeostatic proliferation, signaling defects and reduced functional and metabolic responses, suggesting an exhaustion status. Indeed, the reduced expression of Eomes by BM-NK cells from leukemic mice (**Figure 1C**) is in line with this hypothesis (59, 65). To confirm this hypothesis, we measured the expression of another marker of exhaustion of NK cells, the co-inhibitory molecule TIGIT (66), and we observed a higher frequency of TIGIT positive NK cells in leukemic mice (**Figure 6F**). Altogether, these data indicate that NK cells of leukemic mice are exhausted and suggest an implication of the chronic pro-inflammatory stimulation, partially mediated by IL-15 in the BM.

### NK Cells From AML Patients Display Cues of Metabolic Defects

**Figure 7:**
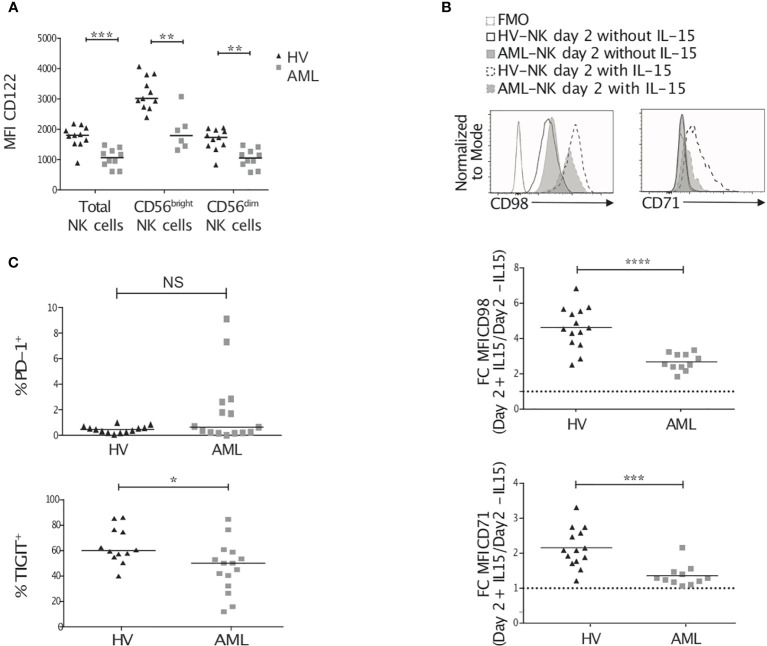
NK cells from AML patients have reduced IL-2/15Rβ and metabolic marker expression. Peripheral blood (PB) NK cells from healthy volunteers and AML patients were analyzed by flow cytometry for CD122 expression **(A)**, CD98 and CD71 surface expression **(B)**, and exhaustion marker expression **(C)**. **(A)** Once thawed up, PB NK cells were stained for CD122, CD3, CD56 and CD45. Data show CD122 expression on CD45^+^/live total NK cells or CD56^bright/dim^ NK cells. **(B)** Peripheral blood mononuclear cells were thawed and incubated for 48h in the presence or absence of 20ng/mL rhIL-15. The histograms show an example of CD98 and CD71 expression on NK cells at day 2 after culture with or without IL-15. The graphs show CD98 (top) and CD71 (bottom) expression ratio (stimulated versus unstimulated). **(C)** The level of expression of PD-1 (top) and TIGIT (bottom) in freshly thawed PB NK cells is depicted. Lines within each group correspond to the median. *p < 0.05, **p < 0.01, ***p < 0.001, ****p < 0.0001, as determined by Mann-Whitney test.

We next sought to determine whether NK cells from AML patients also displayed IL-15 signaling defects and metabolic reduction. We analyzed peripheral blood NK cells from patients diagnosed with AML and compared with healthy donors’ NK cell expression of IL-15Rβ/CD122. We noted a lower surface expression of CD122 on AML patients’ NK cells (**Figure 7A**). in humans, the final steps of NK cell maturation are characterized by the expression of the marker CD56/NCAM defining 2 different maturation subsets: CD56bright representing immature NK cells, which give rise to more mature CD56dim NK cells. Although not all patients had sufficient numbers of CD56^bright^ NK cells, as previously shown (67), we found that both NK cell maturation subsets displayed a reduced CD122 expression in AML patients (**Figure 7A** **)**. Next, we analyzed the expression of CD98 and CD71 as surrogate markers of cellular metabolism (68). In contrast to leukemic mice NK cells, AML patients’ NK cells had similar levels of expression of CD98 and higher levels of expression of CD71 as compared to that of healthy donors (**Supplemental Figure 6**). However, NK cells from AML patients were less able to increase the expression of CD98 and CD71 in response to a stimulation with IL-15 *in vitro* for 48h (**Figure 7B**). Finally, we analyzed the expression of exhaustion markers on NK cells such as PD-1 and TIGIT. While TIGIT expression was slightly lower on AML patients’ NK cells, a fraction of patients’ NK cells expressed high levels of PD-1 (**Figure 7C**).

## Discussion

The mechanisms by which NK cells acquire developmental and functional defects in AML remain mostly unknown. Here, we have studied AML-induced defects in NK cell maturation, homeostatic proliferation and functions in a mouse model that recapitulates human disease. We showed that NK cells of leukemic mice displayed active cytokine signaling while exhibiting alterations in the IL-15/mTOR signaling and metabolism. We also provided evidence that NK cell alterations could be the result of chronic stimulation leading to their exhaustion.

We first observed that the increase in BM immature NK cell frequency paralleled that of the acceleration phase of leukemia progression. The increase in CD27^+^CD11b^—^ NK cells was confirmed in two other mouse models of AML. We also noted a reduction in NK cell functions after stimulation with cancer cell lines or with PMA and ionomycin. In humans, a maturation blockade was found in almost 10% of patients with AML, and patients harboring hypomature NK cells have reduced overall survival and reduced relapse-free survival, in comparison with patients with a normal NK cell profile (29). In addition, functional NK cell exhaustion was previously described in AML patients (36). Indeed, the AhR pathway was shown to induce the expression of the microRNA miR29b in murine and human NK cells in AML. This pathway targets Eomes and T-bet leading to a maturation blockade and functional defects in NK cells (37, 69). In our model, we observed a decrease in the expression of Eomes and T-bet in spleen NK cells and in CD27^+^CD11b^—^ immature NK cells in the BM. However, T-bet expression was not downregulated in CD27^-^CD11b^+^ BM-NK cells. This result might explain the maintenance of CD27^—^CD11b^+^ NK cells which maturation seems to rely mostly on T-bet (52). Furthermore, the AhR-Mir29b pathway did not inhibit the functions of mature human NK cells (69), suggesting that other mechanisms might be responsible of the defects observed in these cells. In our model, NK cell functions were reduced at all NK cell maturation stages. Thus, our data with respect to IL-15-mTOR pathway and metabolism, suggest an alternative pathway for NK cell alterations in AML.

Alternatively, TGF-β is an immunosuppressive cytokine that can dampen NK cell cytotoxicity by *i)* affecting NK cell maturation, *ii)* altering IL-15/mTORC1-dependent signaling and metabolism, and *iii)* participating to a conversion into non-cytotoxic ILC1-like cells (35, 61, 64, 70, 71). Its secretion within the leukemia microenvironment has been puzzling for many years (30, 72–74), notably because the methods for detection were not sufficiently accurate. Since we found alteration in NK cell maturation, functions, IL-15 signaling, and mTOR-dependent metabolism, in addition to a higher frequency of CD49a^+^ NK cells (marker of ILC1, data not shown), we were prompted to analyze the implication of TGF-β in our model. Indeed, we found a higher phosphorylation of SMAD2/3, the signaling mediators of TGF-β, in NK cells from leukemic mice. However, the removal of TGF-β receptor specifically on NK cells definitively ruled out a strong implication of this cytokine in the reduction of IL-15 receptor, the metabolic alterations and most importantly in the reduction of the effector functions of NK cells from leukemic mice. Yet, we cannot exclude a participation of TGF-β in some aspects of NK cells alterations in AML such as their acquisition. of ILC1-like phenotype (35).

Both maturation and triggering of NK cell functions are dependent on cytokines delivered within the microenvironment (14). Amongst them, IL-15 is of prime importance in NK cell biology. Our study revealed an increase in the concentration of IL-15 in the BM of leukemic mice in two different AML models. Consequently, we observed an increase in the activation of the signaling pathways downstream of IL-15 receptor, i.e. the JAK1-3/STAT5 and the mTOR pathways, in addition to an increased expression of activation markers. Moreover, the expression of the β subunit of IL-15 receptor (CD122) was drastically reduced in leukemic mice NK cells as well as in patients. Downregulation of CD122 following ligation with IL-15 has also been shown in previous studies (24, 25, 56, 75) and strongly suggests a ligand-receptor interaction. Altogether, these elements suggest that NK cells are activated by IL-15 in AML, although we cannot exclude the contribution of other cytokines to the activation of NK cells. Indeed, our cytokine array showed an alteration of the cytokine profiles in leukemic BMs with a tendency towards an increase in multiple pro-inflammatory cytokines. Moreover, our RNA sequencing data on immature NK cells revealed that type I interferon signaling and Interleukin signaling are triggered in leukemic mice, suggesting the implication in the activation of NK cells. These observations fit with a recent observation by Crinier et al. showing that NK cells from AML patients displayed a stress and interferon-induced gene signature (34). Interestingly, type I IFN are known to be important for IL-15 production and transpresentation by dendritic cells (19). Here, we have measured the levels of soluble monomeric IL-15 in the bone marrow. Although IL-15 can be detected in a monomeric form, it is mainly transpresented by myeloid cells in complex with IL-15Rα. Soluble forms of IL-15/IL-15Rα can also be detected. Hence, it would be interesting to measure the total levels of IL-15 production in the bone marrow and to determine which cells are responsible for this increased secretion in AML.

Priming of NK cells with IL-15 has been considered to be necessary to lower the threshold for further target cell recognition. However, we showed that despite their activated profile, NK cells from leukemic mice were hyporesponsive to the stimulation with IL-15 *in vitro*. Particularly, short-term stimulation with IL-15 induced less phosphorylation of the ribosomal protein S6 suggesting an impaired mTOR signaling. As an expected consequence, NK cells of leukemic mice exhibited reduced expression of the metabolic markers upon *in vivo* activation of mTOR with poly(I:C). Moreover, metabolic and functional responses of NK cells were also reduced after 24h stimulation with IL-15 *in vitro* (data not shown). Of note, poly(I:C) stimulation increases the production of IL-15 in mice (76). Thus, the decrease in metabolic responses following stimulation with poly(I:C) *in vivo*, or with IL-15 *in vitro*, might be due to the reduced expression of IL-15 receptor subunits leading to reduced overall responsiveness to this cytokine. Yet, we observed reduced expression of nutrient transporters in leukemic mice NK cells, without stimulation by poly(I:C), while mTOR signaling is more activated. These data suggest that the reduced expression of IL-15 receptors is not the only mechanism leading to reduced metabolic responses in leukemic mice NK cells. In AML patients, NK cells also displayed reduced IL-15Rβ/CD122 expression, and although CD71 and CD98 expression was similar to controls at steady state, NK cells from patients showed a limited up-regulation of these metabolic surrogate markers upon cytokine stimulation.

Noteworthy, cellular metabolism is commonly assessed by measurement of oxygen consumption, glycolysis rate, ATP production and respiration, using the Seahorse technology. Indeed, Felices et al. demonstrated that *in vitro* continuous IL-15 treatment reduces primary human NK cell activity through reduction of cellular metabolism (57). However, the number of cells required for such technology exceeds the number of *ex vivo* unexpanded NK cells collected from leukemic mice. Nonetheless, the consistent reduction in all the parameters known to be increased in metabolically active NK cells such as cell size, granularity, the expression of nutrient transporters, their uptake of glucose, their mitochondrial mass and their expression of mitochondrial ROS, strongly suggest that NK cells of AML-bearing mice exhibit metabolic defects (15, 60, 68). The use of refined methods allowing assessment of limited numbers of cells, such as Met-Flow (77) or SCENITH (78) will certainly allow a better understanding of NK cell metabolism in cancer.

Hyporesponsiveness and reduction of functions following prolonged stimulation of NK cells with IL-15 were previously described in humans and in mice and these studies reported signaling and metabolic defects (55, 57–59). Maturation defects were also reported following constitutive activation of the mTOR pathway (79), or continuous *in vivo* treatment with IL-15 (58). Moreover, prolonged activation of NK cells with other cytokines, such as IL-2, IL-12 and type I interferons, or chronic stimulation through activating receptors such as NKG2C, have been associated with deleterious effects (80, 81). Altogether, these studies showed that persistent stimulation can exhaust NK cells. So far, no previous study has associated NK cell exhaustion in cancer condition with persistent stimulation with pro-inflammatory cytokines. Increased serum IL-15 levels, as well as activation of IL-15 and interferon signaling in NK cells were reported in AML patients (34, 82). The assumption that mTOR activation is leading to the exhaustion of NK cells in leukemic mice led us to hypothesize that targeting mTOR pathway would at least reduce some of the observed effects of leukemia progression. Felices et al. showed that treating NK cells with rapamycin, an mTORC1 inhibitor, rescued IL-15-mediated exhaustion of NK cells *in vitro* (57). In leukemic mice, rapamycin induced a partial rescue of NK cell metabolic defects. Moreover, cytokine production after poly(I:C) stimulation was increased in rapamycin-treated NK cells from leukemic mice. Nonetheless, it is important to conclude cautiously on the effects of such treatments in our leukemic mouse model, since mTOR inhibitors likely affect leukemia progression and *per se* reduce the pressure on NK cells.

In mouse, TIGIT, rather than PD-1, has been shown to be a relevant marker for NK cell exhaustion (66). Here, we found that NK cells from leukemic mice had a higher TIGIT expression and we did not detect PD-1 expression (data not shown). In contrast, some AML patients displayed a higher frequency of PD-1 positive NK cells, but slightly lower levels of TIGIT were observed. This discrepancy may reflect the differences in AML progression dynamics in mouse and humans or in the cell surface hallmarks of exhaustion in mouse and human NK cells. Nonetheless, the sum of alterations found in NK cells from leukemic mice and from AML patients (from ours or previous studies) pinpoints a functional exhaustion of NK cells in AML.

During the last two decades, several attempts to enhance NK cell functions as a therapy for the treatment of AML or other cancers have been tested including the stimulation *in vitro* or *in vivo* by γc cytokines (IL-2, IL-15, IL-21, etc.). For instance, recently, Romee R et al. performed the first-in-human clinical trial for the injection of the IL-15 superagonist complex ALT-803 for the treatment of AML patients in relapse (NCT01885897) (83). The phase I clinical study showed that this modified IL-15 is well tolerated and induces an increase in NK cell numbers and markers of functionality (83). However, an increase in the expression of the checkpoint receptors LAG-3 and Tim-3 at the surface of NK cells was described in patients. Other strategies for leukemia treatment are currently studied and performed, based on NK alloreactivity or CAR-NK cells. Although these strategies are very promising, resistance or relapse remain a major concern, and the consequent exhaustion related to NK cell expansion may be involved (84). In light of our data, but also supported by previous work from other research groups, it may be of critical importance to better understand cytokine-induced exhaustion of NK cells. Besides, depending on the time of injection, i.e. during a full blown or relapsed leukemia or after complete remission as prophylactic treatment, it might be relevant to study the impact of leukemia on the NK cells used for such therapies.

In summary, we report here that NK cells in AML are exhausted likely because of a chronic stimulation, in part mediated by the IL-15. These exhaustion traits parallel the alteration of their IL-15 signaling and the reduction of their metabolic and functional responses. Our study provides a new perspective of the mechanisms of NK cell defects in AML that might impact the therapeutic strategies used in this disease.

## Data Availability Statement

The datasets presented in this study can be found in online repositories. The names of the repository/repositories and accession number(s) can be found below: GEO, GSE180409.

## Ethics Statement

The studies involving human participants were reviewed and approved by Institut Paoli-Calmettes Translational Research Committee. Agreement N° LAM-NK2020-IPC 2020-019–20-001. The patients/participants provided their written informed consent to participate in this study. The animal study was reviewed and approved by Committee for Animal Experimentation of Marseille (CAE of Provence number 14), France (2–091009) Study agreement No. 2016041809182209.

## Author Contributions

BB-T designed the study, performed experiments, analyzed the data and wrote the manuscript. NS, VL, SJ-L, PL, JF, RL, and MB performed experiments and analyzed the data. VL and GB performed RNAseq analyses. OH provided FLB1 cells and edited the manuscript. YK performed experiments, analyzed the data and edited the manuscript. TW conceived the research and edited the manuscript. DO, MA-L, and NJA edited the manuscript. CF initiated, conceived, designed, and supervised the research, performed some and supervised all experiments, analyzed the data and wrote the manuscript. All authors contributed to the article and approved the submitted version.

## Funding

This work was supported by institutional grants from the Institut National de la Santé et de la Recherche Médicale (Inserm), Centre National de la Recherche Scientifique (CNRS) and Aix-Marseille Université to CRCM; by the Fondation pour la Recherche Médicale (Equipe FRM DEQ20180339209, Equipe « Immunity & Cancer » for DO). This project was supported by specific grants as the programme “Projets Fondation ARC pour la Recherche sur le Cancer PJA 20191209406” (for NJA), by research funding (for CF) from the Canceropôle Provence-Alpes-Côte d’Azur, GEFLUC-Marseille and SIRIC-Marseille (SIRIC–INCa–DGOS–Inserm 6038). BB-T was supported by a doctoral fellowship from Aix-Marseille Université, the Ligue Nationale contre le Cancer, and by a fellowship from Immunology & Cancer Institute (Marseille), VL is supported by a doctoral fellowship from Aix-Marseille Université, NS is supported by a doctoral fellowship from AP-HM.

## Conflict of Interest

The authors declare that the research was conducted in the absence of any commercial or financial relationships that could be construed as a potential conflict of interest.

## Publisher’s Note

All claims expressed in this article are solely those of the authors and do not necessarily represent those of their affiliated organizations, or those of the publisher, the editors and the reviewers. Any product that may be evaluated in this article, or claim that may be made by its manufacturer, is not guaranteed or endorsed by the publisher.
