# Supplementary material for: Chronic IL-15 Stimulation and Impaired mTOR Signaling and Metabolism in Natural Killer Cells During Acute Myeloid Leukemia
Source: Front Immunol. 2021 Dec 17;12:730970. doi: 10.3389/fimmu.2021.730970 (PMC8718679; doi:10.3389/fimmu.2021.730970)
Supplement: Supplementary file 1 [file Image_1.pdf]

Supplemental Figure 1

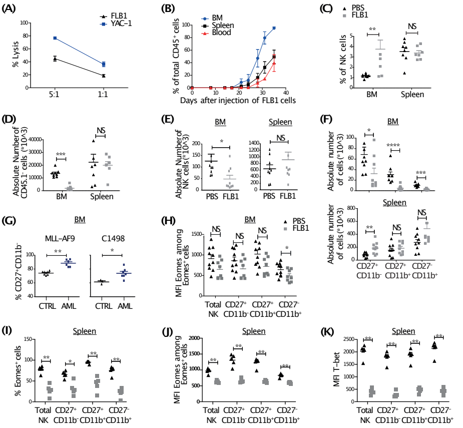

Supplemental Figure 1. NK cell maturation is altered in leukemic mice. (**A**) NK cells were sorted from the spleens of mice and cultured with FLB1 leukemia cells or with YAC-1 target cells for 4h at the indicated Effector:Target (E:T) ratios. The percentage of target cell death was determined by the expression of caspase-3/7 by flow cytometry. The percentage of lysis mediated by NK cells was determined as follow: (% cell death in thepresence of NK cells)- (% cell deathin the absence of NK cells) (n=3 mice).(**B**-**F**, **H**-**K**) C57BL/6 mice were injected intravenously with FLB1 cells. Control mice were injected with PBS. (**B**) The percentage of FLB1 (CD45.1^{+}) cells in relation to total CD45^{+} cells was evaluated by flow cytometry in the bone marrow (BM), spleen and peripheral blood of mice at the indicated days following FLB1 injection (n=2-6 mice/group in 2 independent experiments). (**C**-**F**) Freshly isolated cells from the BM and spleen of leukemic or control mice were stained and analyzed by flow cytometry. (**C**) Percentage of NK cells among total non leukemic CD45.1^{-} cells (n=7-8 mice/group in 2 independent experiments). (**D**-**F**) Absolute number of total non leukemic cells (CD45.1^{-}) (**D**), total NK cells (**E**) or NK cell maturation subsets (**F**) (n=7-9 mice/group in 5 independent experiments). (**G**) The percentage of CD27^{+}CD11b^{-} immature NK cells among total CD3^{-}CD19^{-} NK1.1^{+} NK cellsin the BMof control (CTRL)or leukemic mice(MLL-AF9 transgenic mice or C1498 injected mice) (n=3-8 mice/group in 1 to 4 independent experiments). (**H**) The level of expression of Eomes, as measured by MFI, in Eomes positive NK cells and NK cell maturation subset in the BM of leukemic or control mice (n=8-10 mice/group in two independent experiments). (**I**-**K**) Freshly extracted spleens of leukemic or control mice were stained and analyzed by flow cytometry. The graphs represent the percentage of NK cells expressing Eomes (**I**), the level of expression of Eomes in Eomes positive NK cells (**J**), and the level of expression of T-bet in total NK cells (**K**) (n=5 mice/group).The values are presented as the mean +/- SEM. *p<0.05, **p<0.01,***p<0.001, ****p<0.0001, NS = Non Significant,as determined by Mann-Whitney test.
