# Supplementary figures and images for: Chronic IL-15 Stimulation and Impaired mTOR Signaling and Metabolism in Natural Killer Cells During Acute Myeloid Leukemia
Source: Front Immunol. 2021 Dec 17;12:730970. doi: 10.3389/fimmu.2021.730970 (PMC8718679; doi:10.3389/fimmu.2021.730970)

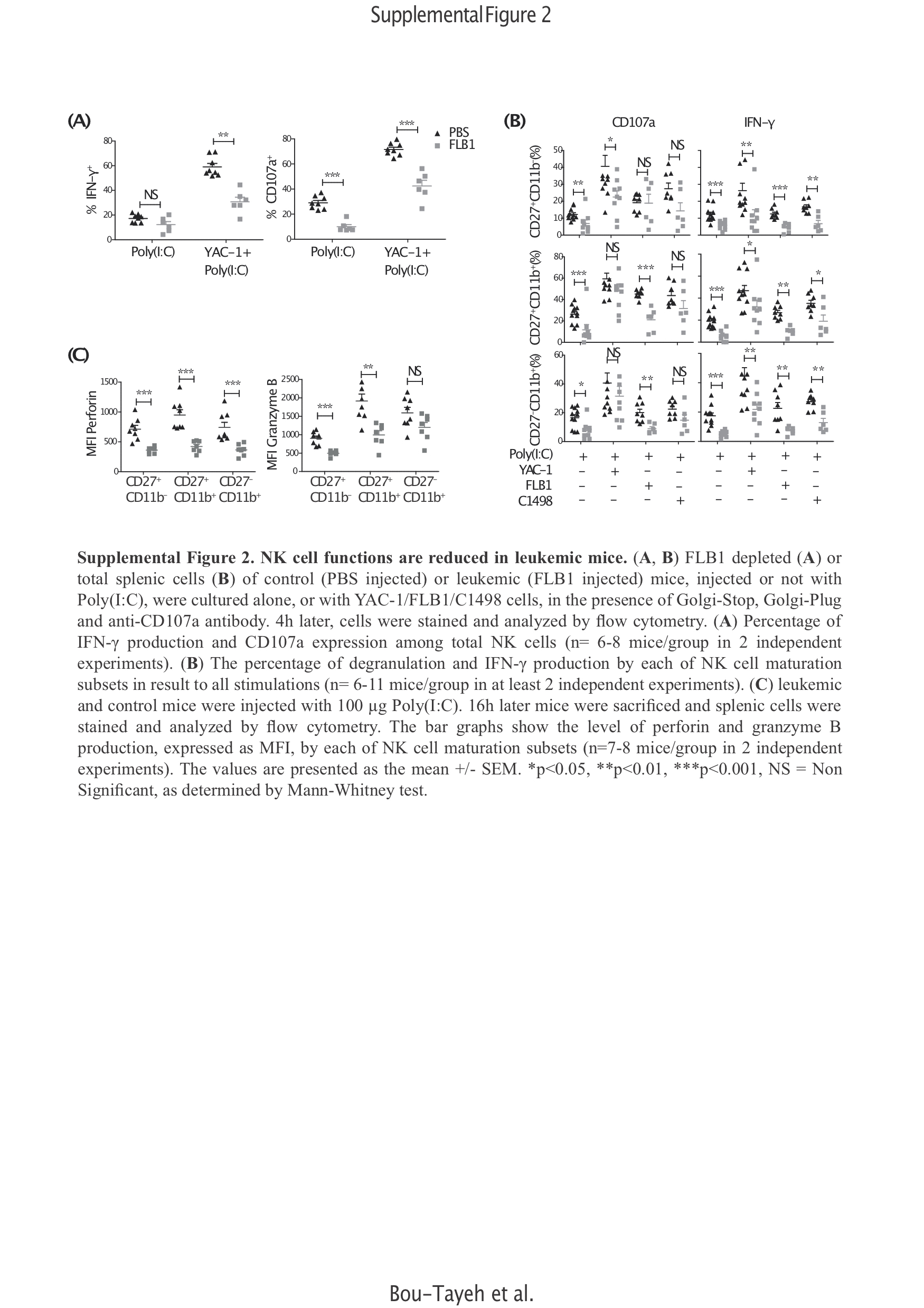

Supplement: Supplementary file 2 [file Image_2.jpeg]

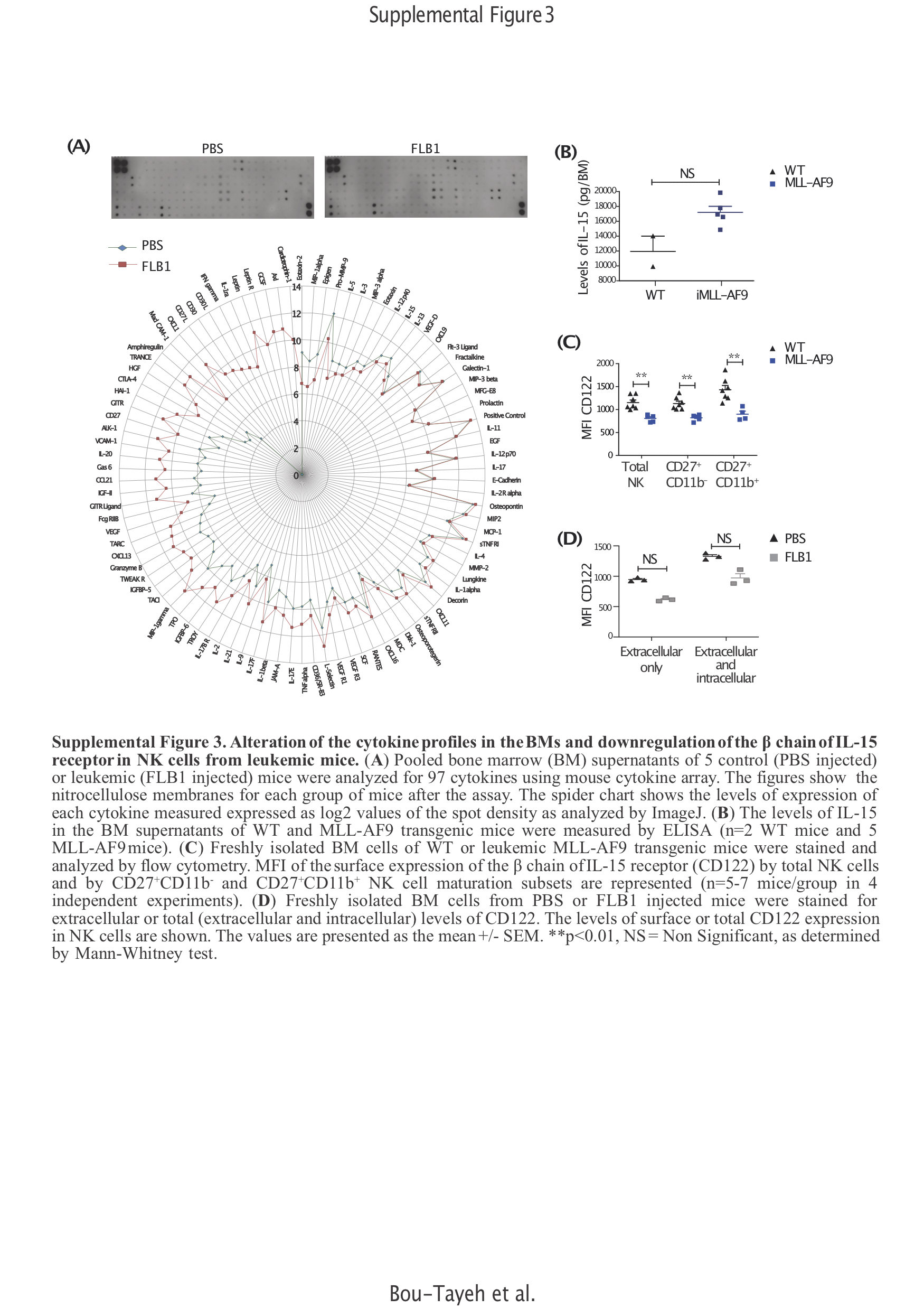

Supplement: Supplementary file 3 [file Image_3.jpeg]

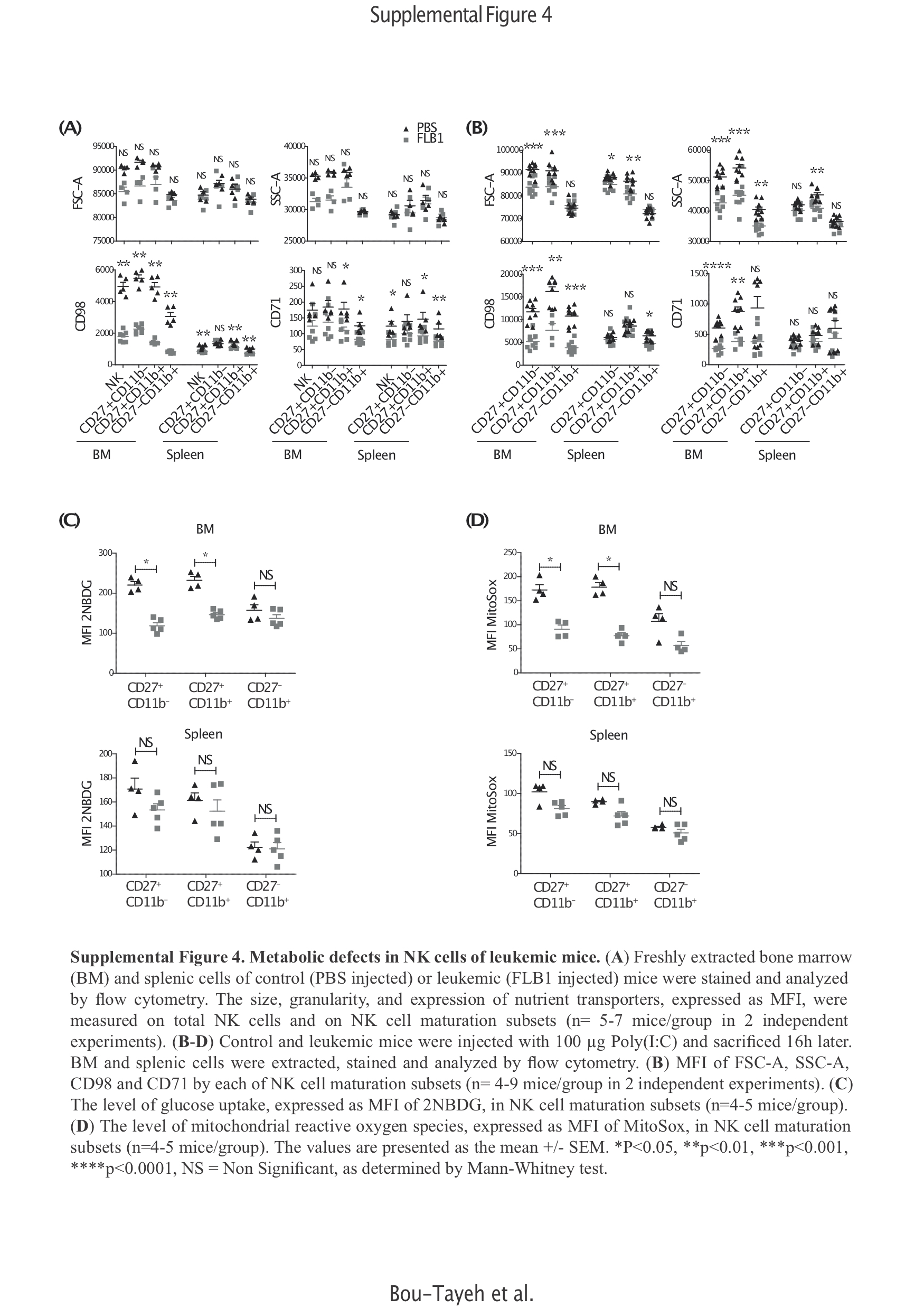

Supplement: Supplementary file 4 [file Image_4.jpeg]

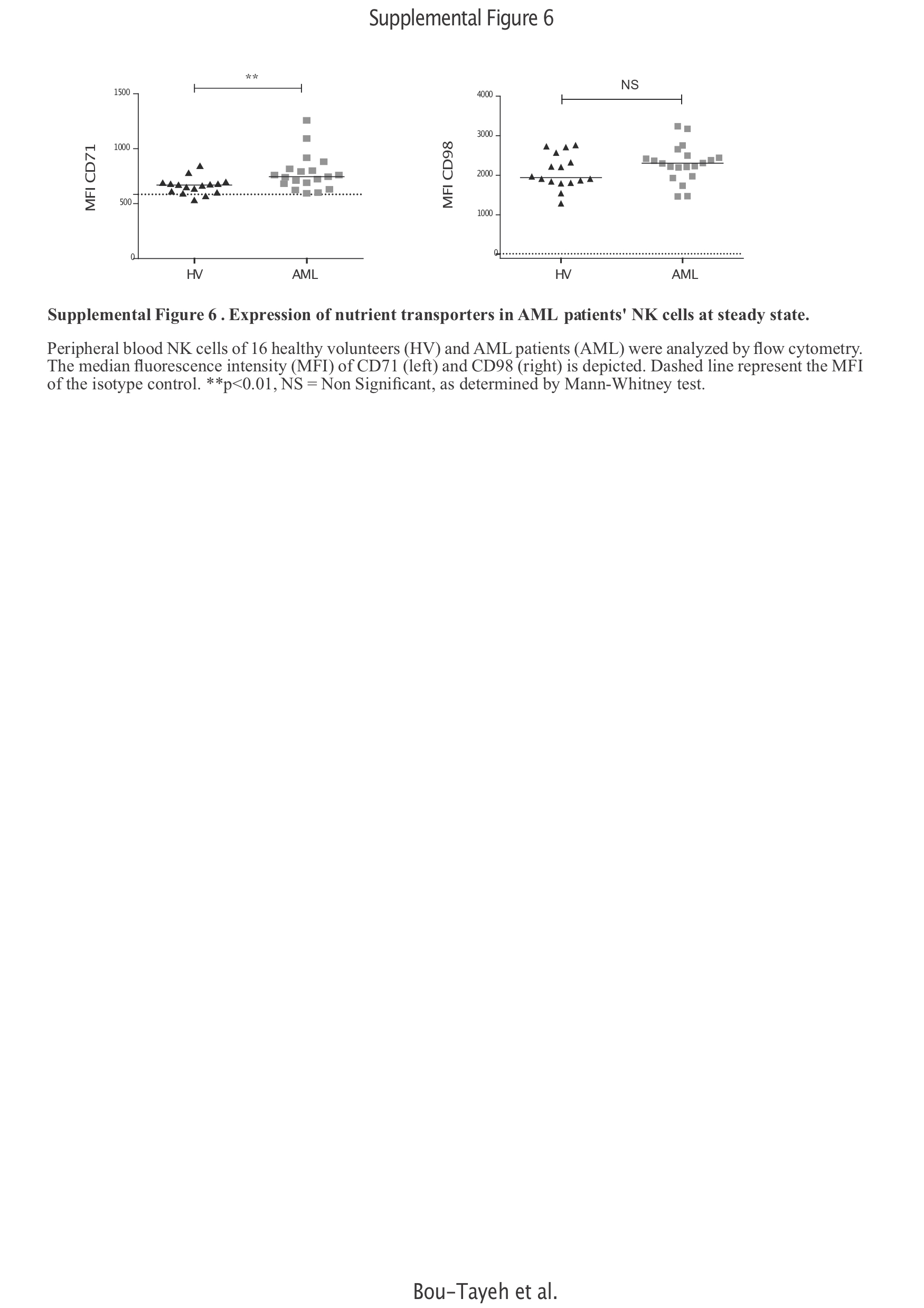

Supplement: Supplementary file 6 [file Image_6.jpeg]
