# Supplementary material for: Chronic IL-15 Stimulation and Impaired mTOR Signaling and Metabolism in Natural Killer Cells During Acute Myeloid Leukemia
Source: Front Immunol. 2021 Dec 17;12:730970. doi: 10.3389/fimmu.2021.730970 (PMC8718679; doi:10.3389/fimmu.2021.730970)
Supplement: Supplementary file 5 [file Image_5.pdf]

Supplemental Figure 5

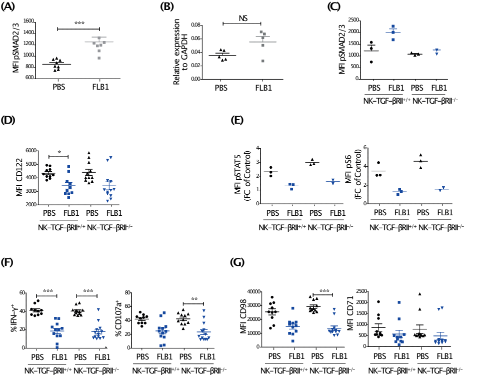

Supplemental Figure 5. Depletion of TGF-β signaling in NK cells from leukemic mice is not sufficient to restore the IL-15 signaling, metabolic and functional defects. (**A**) Freshly isolated bone marrow (BM) cells from leukemic (FLB1 injected) and control (PBS injected) mice were stained and analyzed by flow cytometry. The graph shows the level of expression of phosphorylated SMAD2/3 proteins, as measured by MFI, in total NK cells (n=8 mice/group in 3 independent experiments). (**B**) The level of TGF-β1 expression, relative to control GAPDH expression, was measured by real-time RT-PCR on total BM cells from leukemic or control mice (n=5 mice/group in 2 independent experiments). (**A**, **B**) ***p<0.001, NS= Non Significant, as determined by Mann-Whitney test. (**C**) The level of expression of pSMAD2/3 proteins in total BM NK cells of leukemic and control $Ncr1^{iCre/WT}$ $TGF\beta RII^{WT/WT}$ (NK-TGF-βRII$^{+/+}$) or $Ncr1^{iCre/WT}$ $TGF\beta RII^{fl/fl}$ (NK-TGF-βRII$^{-/-}$) mice (n=2-3 mice/group). (**D**) The level of expression of the β chain of IL-15 receptor (CD122) in total NK cells (n=4-6 mice/group). (**E**) BM cells from leukemic or control NK-TGF-βRII$^{+/+}$ or NK-TGF-βRII$^{-/-}$ mice were cultured with 30ng/mL of IL-15 for 1h. Cells were then stained and analyzed by flow cytometry. MFI of intracellular pSTAT5 and pS6 are given as a ratio of MFI at 30ng/mL IL-15 normalized to the MFI in the absence of IL-15 (n=2-3mice/group). (**F**, **G**) Mice were injected with 100 µg Poly(I:C) and sacrificed 16h later. (**F**) Freshly extracted splenic cells were cultured with YAC-1 target cells in the presence of Golgi-stop, Golgi-plug and anti-CD107α antibody. 4h later cells were stained and analyzed by flow cytometry (n=10-12 mice/group in 3 independent experiments). (**G**) MFI of CD98 and CD71 on total BM NK cells (n=10-12 mice/group in 3 independent experiments). (**D**, **F**, **G**) *p<0.05, **p<0.01, ***p<0.001, NS = No Significant, as determined by Kruskal-Wallis test. The values are presented as the mean +/- SEM.
